# Carbon dots from contaminated *Eichhornia crassipes* roots for spectrally multiplexed identification and sensing of solvents

**DOI:** 10.1039/d6ra00409a

**Published:** 2026-04-28

**Authors:** M. Rangel, S. D. Torres Landa, I. E. Serrato-Mireles, Y. Kumar, N. Dasgupta Schubert, J. E. García, J. S. Pérez-Huerta, V. Agarwal

**Affiliations:** a Centro de Investigación en Ingeniería y Ciencias Aplicadas, IICBA- UAEM, Av. Univ. 1001 Col. Chamilpa Cuernavaca Morelos 62209 Mexico vagarwal@uaem.mx; b Unidad Académica de Ciencia y Tecnología de la Luz y la Materia-UAZ Circuito Marie Curie S/N, Parque de Ciencia y Tecnología Quantum Ciudad del conocimiento 98160 Zacatecas Zac. Mexico; c Facultad de Ciencias Fisicomatemáticas, Universidad Michoacana de San Nicolás de Hidalgo Gral. Francisco J. Mújica S/N. C.U. 58030 Morelia Mich. Mexico; d Facultad de Física y Matemáticas (FCFM), Universidad Autónoma de Nuevo León (UANL), Cd. Universitaria San Nicolás de Los Garza N.L.,66451 Mexico; e Secihti -Cinvestav, Unidad Saltillo Ave. Industria Metalúrgica 1062, Parque Industrial Ramos Arizpe 25900 Coahuila Mexico

## Abstract

Water hyacinth (*Eichhornia crassipes*) is an invasive aquatic plant (originally from South America) that colonizes tropical and subtropical waterbodies worldwide. In this work, we present the sustainable application of carbon dots, obtained by green chemistry from *E. crassipes* roots, as fluorescence-based ratiometric sensors of ethanol and acetone traces in water with a limit of detection (LOD) of ∼1% v/v and ∼0.03% v/v, respectively. The proposed carbon nanoprobe acts as a spectrally multiplexed sensing platform to simultaneously monitor multiple spectral features associated with molecular interactions, allowing for the detection of methanol-spiked ethanol under laboratory conditions with a LOD of ∼1.4% v/v. The viability of the multi-responsive sensor has also been tested in methanol-adulterated commercial alcoholic beverages. The solvation effect of the proposed CDs in different surrounding systems could lead to their potential application as a straightforward, eco-friendly, and affordable alternative for the quality control of certain solvents, thereby improving accuracy, sensitivity, and selectivity compared to traditional analytical methods as well as safeguarding public health by preventing the consumption of adulterated alcoholic beverages.

## Abstract 2

Carbon dots from water hyacinth roots as ratiometric optical sensors for trace levels of acetone, methanol, and ethanol, with practical use in detecting methanol-adulterated alcoholic beverages.

## Graphical abstract

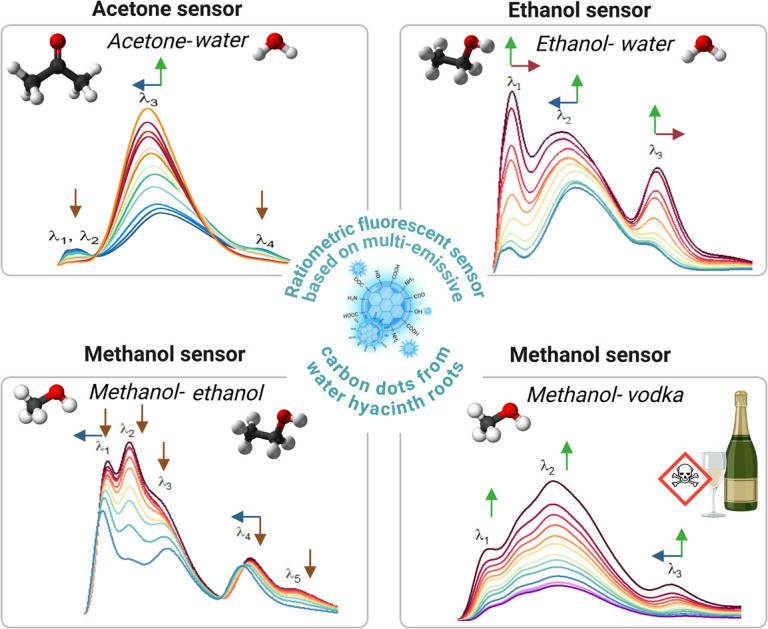

## 1. Introduction

Methanol (methyl alcohol) is commonly used in paints, dyes, and chemical synthesis.^1^ The appearance and odor of methanol and ethanol are highly similar; nevertheless, methanol is toxic to humans. Methanol may find its way into alcoholic beverages through multiple pathways. Methanol is present in traditional alcoholic beverages and can pass through inadequate distillation processes, or be illicitly added to produced liquor, leading to methanol adulterated beverages, which can cause adverse effects on the human body, such as blindness and even death.^2–4^ According to the U.S. Food and Drug Administration (FDA), contamination of alcohol-based hand sanitizers with methanol represents a serious safety concern that increased after COVID-19, as it presents a risk of methanol poisoning.^5^ The main route to intoxication by methanol is through ingestion; however, it can occur by inhalation and skin absorption, causing nausea, vomiting, abdominal pain, impaired consciousness that may progress to severe metabolic acidosis, optic nerve damage, convulsions, coma, and ultimately death.^6,7^

Typical methods to detect methanol require expensive and elaborate techniques such as chromatography, nuclear magnetic resonance (NMR) and Raman spectroscopy.^2,8^ Some efforts to develop new and simple methanol detection techniques include several approaches. Shemirani *et al.* suggested a portable sensor based on the exploitation of chiral nematic liquid crystals and a textile grid for the detection of methanol (0, 2, 4, and 6 wt%) in red wine and vodka.^1^ J. van den Broek *et al.* created a handheld methanol detector based on a separation column-sensor concept. The column is a small packed bed of polymer adsorbent (Tenax TA), which separates methanol from ethanol, and then methanol is detected by a nanostructured Pd-doped SnO_2_ gas sensor. The authors analyzed liquor spiked with methanol and quantified 0.3% v/v. of methanol.^9^ J. Barroso *et al.* measured methanol traces in alcoholic beverages through fluorescence spectroscopy and photoelectrochemical (PEC) analysis. Their analytical system is based on the oxidation of cysteine (CSH) with hydrogen peroxide (H_2_O_2_).^10^ Undesirable biomass-derived carbon dots (CDs) exhibit notable properties, such as tunable fluorescence, high solubility and eco-friendliness, which make them suitable for use in optical sensors.^11–13^ The interactions between CDs and the surrounding solvent environment significantly influence their physicochemical properties and sensing performance.^14,15^ CDs from water hyacinth (*Eichhornia crassipes*) leaves modified with thiol groups are excellent nanoprobes for detecting metal ions in aqueous medium.^16^ The pesticide pretilachlor was detected using CDs derived from *E. crassipes* leaves as a precursor.^17^ CDs derived from the leaves of *E. crassipes* and composited with copper sulfide nanoparticles were synthesized for the degradation of Brilliant Green dye.^18^ Water hyacinth is considered an aggressive and invasive aquatic plant that forms dense mats, reduces the oxygen level of native aquatic species, and hosts organisms that transmit infections to humans.^19,20^ The rapid proliferation of their fibrous roots can block the transport of water, with severe economic and environmental consequences.^21^

In this work, we prepared fluorescent CDs derived from water hyacinth roots, obtained from a heavy metal ion-contaminated lagoon, by the simple carbonization method. Fluorescent CDs were employed as ratiometric sensors that exhibited multi-response capabilities for detecting trace amounts of acetone and ethanol in water, as well as methanol in ethanol. Moreover, they enabled quantification of methanol concentrations in adulterated vodka through photoluminescence (PL) wavelength shifts and/or intensity changes, resulting from interactions between the solvent environment and surface functional groups on the CDs.

## 2. Experimental section

### 2.1. Materials and techniques

Water hyacinth roots were collected from Jovita lagoon in Naranja de Tapia, Mich. (19.7757729,-101.7599842). Methanol (99%) was purchased from Sigma Aldrich. Ethanol (99%) and acetone (99.5%) were purchased from Fermont. Vodka Absolut Raspberry (38% alcohol) was used to detect methanol.

For the CD characterization, a Thermo Fisher Scientific Evo-60 spectrophotometer was used to measure absorbance, and PL spectra were obtained using a Cary Eclipse Fluorescence Spectrophotometer. FTIR spectra were acquired with a Varian 660-IR FT-IR spectrophotometer. X-ray diffraction (XRD) patterns were recorded with a Bruker AXS D-8 Advance diffractometer using a LYNXEYE detector. Zeta-potential measurements were recorded using a Malvern Zetasizer Nano series ZEN 3600. A Thermo Fisher Scientific K-Alpha was used for the X-ray photoelectron spectroscopy (XPS) measurements. CDs were analyzed by ^1^H NMR spectroscopy with a Bruker Avance III HD 500 MHz spectrometer; deuterated methanol (CD_3_OD) and deuterated ethanol (C_2_D_3_OD) were used as the solvent. An FE-SEM (Hitachi S5500) microscope was used to visualize the CDs particles. Images of the CDs were captured by transmission electron microscopy (TEM) with an FEI model Talos F200.

Water from the Jovita lagoon, the *E. crassipes* roots, and the as-prepared CDs were analyzed using Bruker Total Reflection X-ray Fluorescence (TXRF) S2 PICOFOX equipment to measure trace amounts of heavy metal ions. The pH, conductivity, and total dissolved solids (TDS) were determined with an Extech EC500 meter. A Hach company DR/820 portable colorimeter was used to detect nitrogen and phosphorus in the *E. crassipes* roots, and its thermogravimetric analysis was carried out with STA PT 1600 equipment. APHA, AWWA & WEF (Standard Methods for the Examination of Water and Wastewater) test methods were implemented to examine the Jovita polluted water quality parameters.^22^ The alkalinity and hardness of the water samples were measured by the titrimetric method.

### 2.2. Procedures

#### 2.2.1. Synthesis of CDs

Water hyacinth roots were used as precursors to obtain CDs by the direct carbonization method at 250 °C for 2 h. Briefly, the roots were washed with deionized water and ethanol, dried for 1 h (60 °C), ground in a coffee mill, and carbonized in a muffle furnace. CDs were dispersed at a concentration of 10 mg mL^−1^ in water (CDs-w) or ethanol (CDs-EtOH), then sonicated for 4 h, centrifuged for 20 min at 13 000 rpm, and filtered through filter paper.

#### 2.2.2. Sensing experiments

CDs-w were used as fluorometric probes to detect trace amounts of acetone and ethanol in water after spiking the samples with varying concentrations of the analytes. The obtained CDs-w were dispersed in the corresponding solution at a concentration of 1% v/v. The detection of methanol traces was achieved by incorporating CDs-EtOH into pure methanol, pure ethanol, or their mixtures, with varying methanol volume percentages, followed by a measurement of their PL emission at an excitation wavelength of 260 nm. 10% v/v of CDs-EtOH was dispersed in the corresponding solution.

## 3. Results and discussion

### 3.1. CDs precursor

**Fig. 1:**
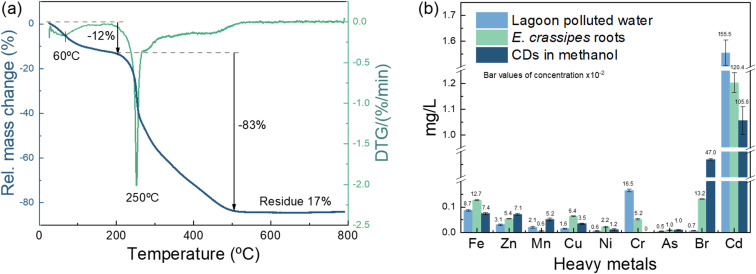
(a) TGA analysis of *E. crassipes* roots, and (b) heavy metals content in the roots and the lagoon water from which *E. crassipes* were collected, as determined by TXRF analysis.

Elemental trace contamination, stability and decomposition of molecules in the natural polymers of the contaminated *E. crassipes* roots as CDs precursors were tested by TGA and TXRF. For TGA, with a heating rate of 10 °C min^−1^, the active pyrolytic zone for *E. crassipes roots* corresponds to a range of 200 to 500 °C (Fig. 1a). The sample showed a mass loss of ∼12% during which moisture and low-molecular-weight compounds were eliminated from ∼25 to 200 °C, degradation of polymers, such as hemicellulose, cellulose, and pectin, and partial degradation of lignin, occurs between 200-500 °C (mass loss of ∼83%). After 500 °C, lignin is not completely degraded; instead, a residual mass of approximately 17% remains as carbon black and ash.^21^ The results of TXRF analysis (Fig. 1b) reveal the presence of some highly toxic heavy metals in the *E. crassipes* roots, in the polluted lagoon water in which they were grown, and in the processed CDs. Some water quality parameters of samples from the Jovita lagoon in Naranja de Tapia, Michoacán, México, and the *E. crassipes* roots, as well as their micro and macronutrients detected by TXRF, are shown in Table S1 and S2.

### 3.2. CDs characterization

**Fig. 2:**
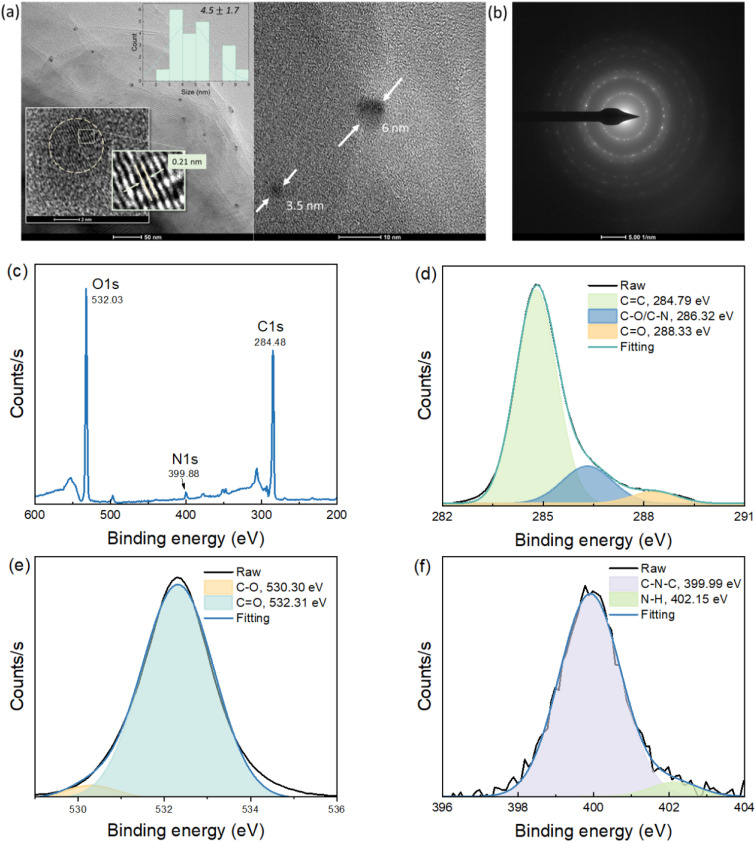
(a) TEM (insets: histogram of size distribution of 20 particles and *d*-spacing magnification) and (b) SAED images of CDs-w. (c) XPS survey scan spectra and XPS high-resolution spectra of (d) C 1s, (e) O 1s, and (f) N 1s.

TEM images of the CDs-w (Fig. 2a) show spherical shapes with a diameter of ∼4.5 ± 1.7 nm and planar lattice fringes with a *d*-spacing of 0. 21 nm related to the (100) plane of the graphitic crystal structure.^23^Fig. 2b shows the SAED pattern of the CDs, which reveals distinct diffraction rings characteristic of a semicrystalline structure. This observation confirms that the prepared CDs possess both ordered crystalline domains and disordered amorphous regions, highlighting their semicrystalline nature.^24^ To elucidate the elemental composition, the CDs were analyzed by XPS. The XPS survey spectrum (Fig. 2c) displays three main peaks corresponding to O 1s (532.03 eV), N 1s (399.88 eV), and C 1s (248.48 eV). The high-resolution (HR) C 1s spectrum was deconvoluted into three signals for C=C (284.79 eV), C–O/C–N (286.32 eV) and C=O (288.33 eV) bonds (Fig. 2d).^25,26^Fig. 2e shows the HR XPS O 1 s spectrum with two deconvoluted bands for the C–O and C=O chemical bonds at 230.30 and 232.31 eV, respectively.^27,28^ The N 1s spectrum (Fig. 2f) reveals two bands designated as the C–N–C and N–H bonds at 399.99 and 402.15 eV, respectively.^29^ The results confirm the presence of enriched functional groups on the surface of the CDs.

**Fig. 3:**
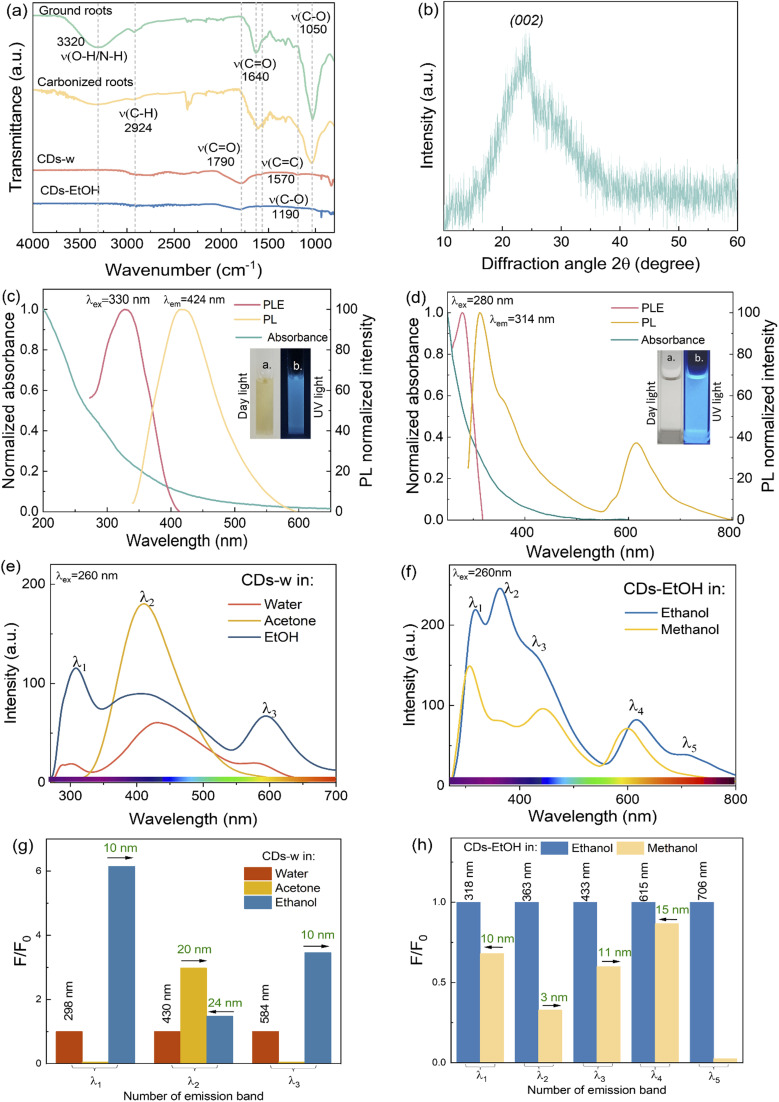
(a) FTIR spectra of ground, carbonized roots, CDs-w and CDs-EtOH. (b) XRD diffraction pattern of the powder carbonized material. (c) PL spectra of CDs-w in water at maximum emission wavelength, UV-vis, and PLE spectra (Inset: Images of CDs-w under day and UV light, 365 nm). (d) UV-vis and PL spectra at maximum emission wavelength, and PLE spectra of CDs-EtOH (Inset: Images of CDs-EtOH under day and UV light, 365 nm). (e) PL spectra of CDs-w in different solvents (1% v/v). (f) PL spectra of CDs-EtOH dissolved in ethanol or methanol (10% v/v). (g) and (h) correspond to the *F*/*F*_0_ ratio of each emission band for CDs-w and CDs-EtOH, respectively (arrows indicate the shift of the maximum emission band with respect to the emission in water for CDs-w and with respect to ethanol for CDs-EtOH). *F*_0_ is the fluorescence of the reference (CDs-w in water or CDs-EtOH in ethanol), and *F* is the fluorescence of CDs in other solvents.

FT-IR analysis was performed on ground roots (light green), carbonized roots (yellow), CDs-w (orange) and CDs-EtOH (blue) (Fig. 3a). The ground and carbonized root samples exhibit a broad peak at ∼3240 cm^−1^, indicative of –OH/NH stretching, alongside a band at ∼2924 cm^−1^ attributed to the stretching vibration of the C–H bond.^30,31^ The presence of hydroxyl and amine groups enhances the hydrophilicity, stability, and dispersibility of CDs in water, enabling their use in various applications such as sensing and bioimaging. The bands at ∼1640 cm^−1^ and ∼1054 cm^−1^ suggest the presence of a stretching vibration of the C=O double bond and C–O stretching.^31–33^ Compared to the ground roots, the broad peak intensities at ∼3240 cm^−1^, ∼1640 cm^−1^, and ∼1050 cm^−1^ are reduced following carbonization. For CDs-w and CDs-EtOH, the stretching vibrations of the C=O double bond and C–O stretching are displaced to ∼1790 cm^−1^ and ∼1190 cm^−1^, respectively. The intensity of the C=O signal is higher for CDs-w than for CDs-EtOH.

X-ray diffraction (XRD) pattern of the CDs (Fig. 3b) showed a single broad peak centered at 2*θ* = 23.0°, which is consistent with the (002) lattice spacing of carbon-based materials with abundant sp^3^ disorder.^34^ The presence of broad peaks distinguishes the carbon quantum dots from graphene and graphite, since the graphene and graphite show sharper peaks in the XRD pattern.^35^ The zeta potential of CDs-w was measured to be −23.6 ± 5.96 mV, indicating that the surface of the CDs carries a significant negative charge. This negative potential suggests the presence of abundant anionic functional groups, such as carboxyl and hydroxyl moieties, on the CDs surface. The moderately high absolute value of the zeta potential reflects good colloidal stability, as the electrostatic repulsion between particles helps to prevent aggregation. The zeta potential of CDs-EtOH is 1.46 ± 0.21 mV, indicating that the particles are typically characterized as nearly neutral and exhibit a pronounced tendency toward aggregation. The FE-SEM image of CDs-w revealed semispherical particles with an average diameter of 61 ± 22 nm (based on 20 particles), along with noticeable agglomerations (Fig. S1). A wide band can be observed in the UV-vis absorption spectrum (Fig. 3c) without any apparent characteristic peak for CDs-w. The weak peaks observed at 300 and 260 nm are attributed to the *n*–π* transition of C=O and the π–π* transition of C=C within a conjugated system, respectively.^36^ The PL of CDs-w is strongly dependent on the excitation wavelength (Fig. S2). The fluorescence spectrum demonstrates that when excited at a wavelength of 330 nm (the wavelength of excitation that yields the highest emission intensity), the CDs-w shows an emission peak at 424 nm. As seen in the inset, CDs-w exhibits a blue color when exposed to UV light and a light-brown color when exposed to daylight. PL at an excitation wavelength of 280 nm (excitation that yields the highest emission intensity), PLE, absorbance, and CDs-EtOH images under natural and UV-light are included in Fig. 3d.

**Fig. 4:**
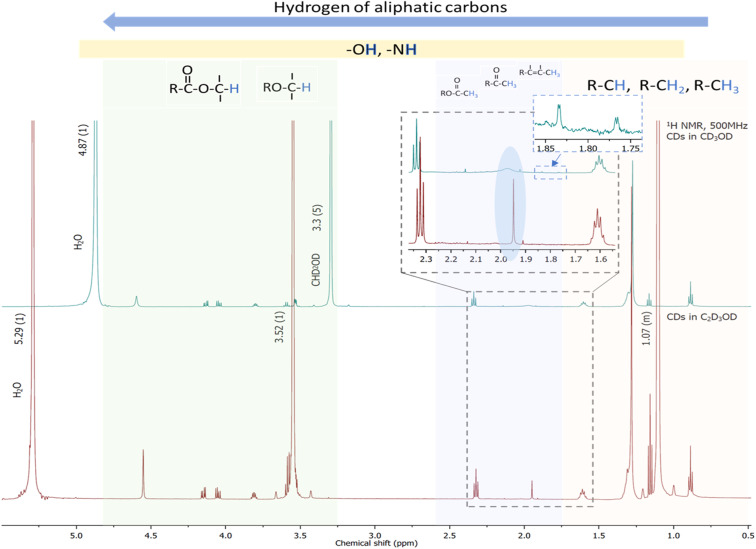
^1^H NMR spectra of CDs dispersed in deuterated methanol (residual signals of solvent: 4.87 ppm from deuterated water and 3.3 ppm from the proton of CHD_2_OD) and deuterated ethanol (residual signals of solvent: 5.29 ppm from deuterated water, 3.51 ppm from protons of CH_2_ and 1.07 from protons in CH_3_). The insets highlight the zoomed region from 2.4 to 1.5 ppm, emphasizing the main differences between the two spectra.

^1^H NMR spectroscopy serves as a powerful analytical tool for probing the chemical environment of hydrogen atoms associated with the surface functional groups of CDs. It provides detailed insights into their surface chemistry and can reveal solvent-induced changes in functional group interactions and dynamics.^37^Fig. 4 shows the ^1^H NMR spectrum of CDs derived from *E. crassipes* roots in deuterated methanol and in deuterated ethanol. Most of the signals are slightly shifted in deuterated ethanol relative to those in deuterated methanol. These shifts arise primarily from variations in the local electronic environment surrounding hydrogen nuclei, which influence the degree of shielding or deshielding experienced by each proton. Nevertheless, notable spectral changes were observed in the spectrum of CDs in CD_3_OD, including a broad signal at 1.98 ppm, probably related to an amine group and two doublets at 1.83 and 1.76 ppm, possibly from complex couplings. In contrast, the spectrum in C_2_D_5_OD exhibits only a sharp peak at 2.35 ppm in the corresponding region. Spectral differences observed in the ^1^H NMR analysis confirm that the solvent environment has a significant influence on the structural features of CDs.^38^ These solvent-dependent variations emphasize the dynamic nature of CDs' surface chemistry, demonstrating that functional groups are highly responsive to changes in solvent polarity and hydrogen-bonding capacity. Such findings highlight the critical role of solvent interactions in modulating the behavior of surface functionalities, with direct implications for the stability, reactivity, and range of applications of CDs.

This pronounced difference between the two solvents provides clear evidence that the molecular interactions of CDs are strongly modulated by the solvent environment. Specifically, CD_3_OD appears to promote broadened and more intricate coupling behavior, whereas C_2_D_5_OD stabilizes a simpler, more uniform proton environment. These findings highlight the critical influence of solvent choice on the electronic and structural properties of CDs.

The stability of the as-prepared CDs in the presence of NaCl and at different pH values was investigated by measuring their PL spectra at an excitation wavelength of 330 nm (corresponding to the maximum intensity of emission) (Fig. S3). NaCl concentrations from 0.5 to 4 M have a negligible effect on the PL intensity of CDs-w. At pH 1 and 14, the shift in the PL emission was ∼16 nm to the blue region, while the PL intensity was found to decrease by ∼10% for CDs-w at pH 1 and ∼3% at pH 14 (with respect to pH 7). In the case of CDs-w at pH 2 and pH 13, the maximum emission was shifted ∼10 nm to the blue and was accompanied by an increase in the PL intensity of ∼6% at pH 2 and ∼11% at pH 13. Within the pH range of 3 to 12, the photoluminescence properties of CDs-w remain largely unaffected.

### 3.3. Solvent effect on the PL of CDs

The fluorescence of CDs in solvents of different polarities was examined. Fig. 3e illustrates the difference in PL emission spectra of CDs-w dispersed in three different media, water, ethanol or acetone, under an excitation wavelength of 260 nm. Fig. 3f shows the PL bands of CDs-EtOH in ethanol or methanol, at the same excitation wavelength (260 nm). The CDs-w exhibit solvent-specific photoluminescence responses, enabling the discrimination of the three transparent and mutually miscible solvents: water, acetone, and ethanol. Similarly, dispersions of CDs-EtOH in ethanol or methanol display five distinct response points, enabling differentiation between methanol and ethanol. As illustrated in Fig. 3g, the normalized fluorescence intensity (*F*/*F*_0_) across the three characteristic emission bands of CDs-w reveals that, upon exposure to acetone, only a single emission band persists, indicating selective quenching or spectral suppression of the remaining bands. Furthermore, the identification of water and ethanol is facilitated by distinct spectral shifts observed in the emission profile of CDs-w in ethanol: a 10 nm shift toward lower energies in the first emission band, a 24 nm shift toward higher energies in the second band, and a 10 nm redshift in the third band. For CDs-EtOH, the distinction between ethanol and methanol can be achieved based on both spectral shifts and intensity variations observed in the first four emission bands. Additionally, the complete absence of the fifth emission band in methanol, contrasting with its presence in ethanol, provides a clear spectral signature for solvent discrimination, as illustrated in Fig. 3h. The observed differences in the multi-emission responses of CDs provide the basis for an optical sensor with high accuracy, enhanced reliability, and reduced susceptibility to interference.

The PL behavior of CDs is closely related to the surface states. Previous studies indicate that the solvent-dependent properties of CDs arise from variations in interactions between CDs and the solvent. In polar solvents, hydrogen-bond and dipole–dipole interactions are predominant.^39–41^ It can be inferred that interactions between CDs and solvents of varying polarity result in distinct surface states of CDs, which subsequently influence the energy gaps between the highest occupied molecular orbital (HOMO) and the lowest unoccupied molecular orbital (LUMO), thereby enabling tunable PL.^41^ The clear differences in the emission spectrum of CDs enable their use in solvent identification and quantification.

#### 3.3.1. Detection of acetone traces in water

**Fig. 5:**
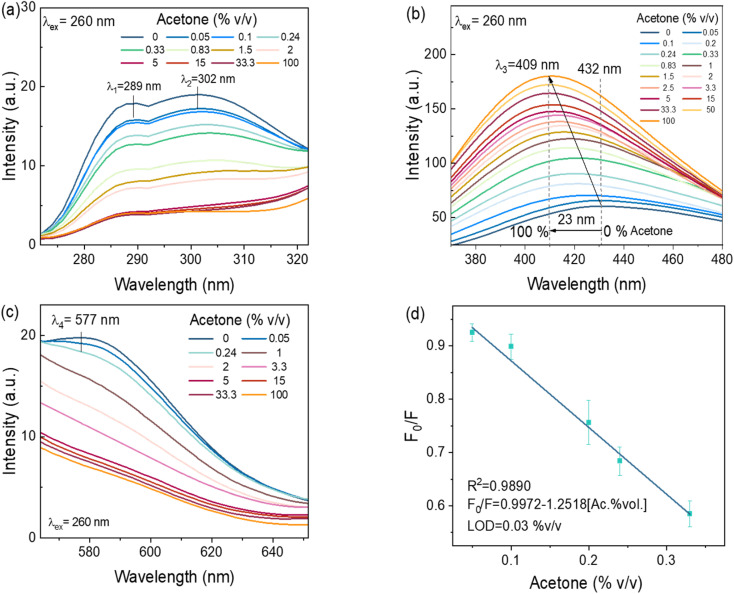
PL multi-response of CDs-w towards different %v/v of acetone in water under an excitation wavelength of 260 nm, emission bands at (a) ∼289 and ∼302 nm, (b) ∼432 to 409 nm, and (c) ∼577 nm, and (d) linear adjustment of *F*_0_/*F* as a function of the concentration of acetone (where *F*_0_ and *F* are the PL intensities of CDs-w in the absence and presence of acetone, respectively) estimated in the emission range of 370–480 nm. Error bars correspond to the standard deviation of three samples.

The multi-emissive CDs-w sensitivity response towards acetone (in deionized water) at an excitation wavelength of 260 nm was examined in a concentration range of 0.05 to 100% v/v (Fig. 5a–c). Fig. 5a illustrates two emission peaks at 289 nm (*λ*_1_) and 302 nm (*λ*_2_), which diminish gradually as the acetone concentration increases. The emission band at 302 nm seems to flatten when the acetone concentration exceeds 5% v/v. The PL peak emission at 432 nm (*λ*_3_) (Fig. 5b) increases gradually with increasing acetone concentration, reaching a maximum of 100% v/v, accompanied by a blueshift of 23 nm. In addition, the shoulder peak at 577 (*λ*_4_) nm exhibits a gradual decline in signal, resulting in the peak's disappearance and changes to a lineal flat line signal (Fig. 5c). To calculate the limit of detection (LOD), the linear range was observed in the emission band corresponding to the region of 370–480 nm (Fig. 5d). The data revealed a LOD of 0.03% v/v of acetone in water, with a coefficient of determination of 0.99. The Stern–Volmer equation was determined to be *F*_0_/*F* = 0.9972 – 1.2518[acetone %*v*].

#### 3.3.2. Detection of ethanol traces in water

**Fig. 6:**
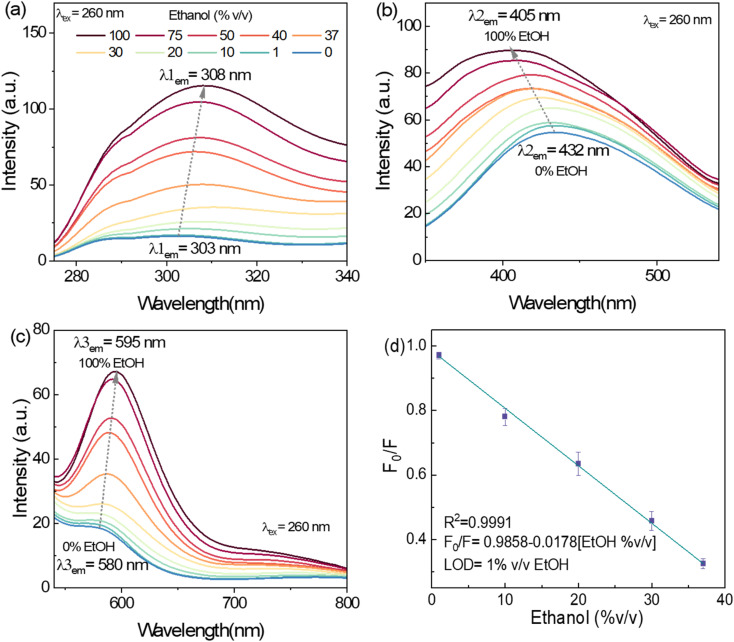
Multi-response of CDs-w towards different %v/v of ethanol in water (excitation wavelength of 260 nm) in three different emission bands at (a) 303 nm, (b) 432 nm and (c) 580 nm and (d) linear adjustment of *F*_0_/*F* as a function of the concentration of ethanol (where *F*_0_ and *F* are the PL intensities of CDs-w in the absence and presence of ethanol, respectively) estimated in the emission range of 275–340 nm. Error bars correspond to the standard deviation of three samples.

CDs-w demonstrated the capability to detect ethanol content in aqueous mixtures, revealing a triple-point response. Fig. 6a displays the emission of CDs-w within the 275–340 nm range, featuring a maximum peak at 303 nm (*λ*_1_). This peak exhibits a 5 nm redshift and increased intensity as ethanol concentration increases from 1 to 100% v/v. The emission in the range of 350–540 nm displays a maximum centered at 432 nm that is displaced to 405 nm (*λ*_2_) as the ethanol concentration increases (Fig. 6b). A third band at 580 nm (*λ*_3_) is shifted 15 nm to the red emission, accompanied by an increase in the PL intensity (Fig. 6c). The LOD was determined in the 275–340 nm range as 1% v/v of ethanol with *R*^2^ = 0.99, and the Stern–Volmer equation is *F*_0_/*F* = 0.9858 – 0.0178[ethanol %v/v] (Fig. 6d).

#### 3.3.3. Detection of methanol traces in ethanol

**Fig. 7:**
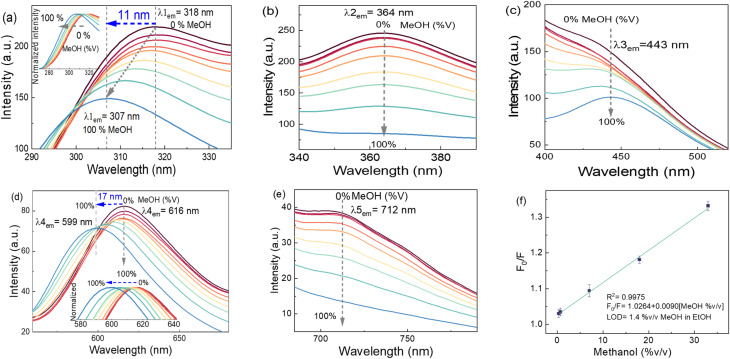
Effect on PL bands of different MeOH concentrations (%v/v) mixed with ethanol using CDs-EtOH (excitation wavelength of 260 nm), multi-response with maximum emission bands at (a) 318 nm (inset shows the normalized PL), (b) 364 nm, (c) 443 nm, (d) 616 nm (inset shows the normalized PL) and (e) 712 nm. (f) Linear adjustment of F_0_/F as a function of the concentration of methanol (where *F*_0_ and *F* are the maximum PL intensities of CDs-EtOH in the absence and presence of methanol, respectively, under excitation at 260 nm, and estimated in the emission range of 340–490 nm. Error bars correspond to the standard deviation of three samples.

In the case of methanol traces in ethanol, at an excitation wavelength of 260 nm, the multi-response CDs-EtOH displays PL spectra with a maximum emission as a shoulder at 318 nm (*λ*_1_), accompanied by shoulders at 443 nm (*λ*_3_) and 712 nm (*λ*_5_), as well as Gaussian peaks at 364 nm (*λ*_2_) and 616 nm (*λ*_4_) (Fig. 7a–e). The results reveal that the incremental addition of MeOH (0 to 100% v/v) to EtOH, at an excitation wavelength of 260 nm, leads to a gradual decrease of the shoulder emission bands at 318 nm (*λ*_1_) and 616 nm (*λ*_4_), along with blueshifts of 11 nm (from 318 to 307 nm) and 17 nm (from 616 to 599 nm), respectively (Fig. 7a and d). Furthermore, it is noted that the shoulder emission at 318 nm systematically transitions to a Gaussian peak with increasing MeOH concentration (Fig. 7a). A comparable observation occurred at the shoulder emission of 443 nm, which converts into a Gaussian peak without a shift in the peak position (Fig. 7c). The emission peak at 364 nm, nearly Gaussian, transitions to a horizontal flat line as the concentration reaches 100% (Fig. 7b), in contrast to the shoulder peaks observed at 318 and 443 nm. The inset (Fig. 7a and d) presents both emissions' normalized spectra and a shift for enhanced clarity. A different observation was recorded at the Gaussian-type shoulder at 712 nm (*λ*_5_), gradually transforming into a linear flat line as the concentration approached 100% (Fig. 7e). The LOD of methanol in ethanol solution is 1.4% v/v, calculated in the emission range of 340–490 nm (Fig. 7f). The coefficient of determination is 0.99, and the Stern/Volmer equation is *F*_0_/*F* = 1.0264 + 0.0090[MeOH %v/v].

#### 3.3.4. Detection of methanol traces in vodka

**Fig. 8:**
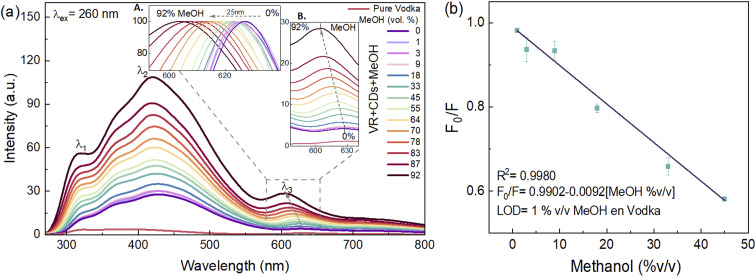
(a) Effect on PL bands of MeOH concentration (1% to 92%v/v) mixed with raspberry vodka using CDs-EtOH (excitation wavelength of 260 nm), response for the maximum emission bands at 316 nm, 420 nm, and 628 nm (inset A shows the normalized PL bands of *λ*_3_; inset B is the zoomed region of *λ*_3_). (b) Linear adjustment of *F*_0_/*F* as a function of the concentration of methanol (where *F*_0_ and *F* are the PL intensities of CDs-EtOH in the absence and presence of methanol, respectively) estimated in the emission range of 350–500 nm. Error bars correspond to the standard deviation of three samples.

Vodka is considered to be a neutral spirit mainly derived from the fermentation and distillation of grain, potatoes, sugar beet, grapes, or cassava.^42^ Vodka composition is high-quality water and ethanol.^43^,^44^ The ability of CDs-EtOH to detect trace amounts of methanol was investigated in vodka berry-natural flavored and samples adulterated with methanol. Fig. 8a shows the PL spectra of CDs-EtOH, at an excitation of 260 nm, for the adulterated alcoholic beverage, which displays three main peaks (316, 420 and 628 nm). The emission band at 628 nm (*λ*_3_) increases in intensity and is shifted 25 nm to the blue emission wavelength (from 0% to 92% v/v of MeOH). Using CDs-EtOH, methanol was detected with a LOD of 1% v/v based on 3*σ*/*k* (where *σ* is the standard deviation of the corrected blank signal and *k* is the slope of the calibration plot).^45^ The linear range (1–45% v/v of methanol) was given by the Stern–Volmer equation of *F*_0_/*F* = 0.9902–0.0092[MeOH %v/v], and *R*^2^ = 0.99 in the maximum emission band (*λ*_2_) at 420 nm (Fig. 8b).

The proposed multi-emissive sensors represent a significant advancement in optical detection, as their multiple emission pathways substantially improve measurement reliability in complex matrices. Unlike conventional single-signal fluorescent probes, which often suffer from limited accuracy and high susceptibility to background interference, multi-emissive systems provide enhanced spectral resolution and signal discrimination. By integrating several emission channels, these intelligent optical sensors achieve superior sensitivity, minimize non-specific responses, and deliver more robust analytical performance.

#### 3.3.5. Possible mechanism

**Fig. 9:**
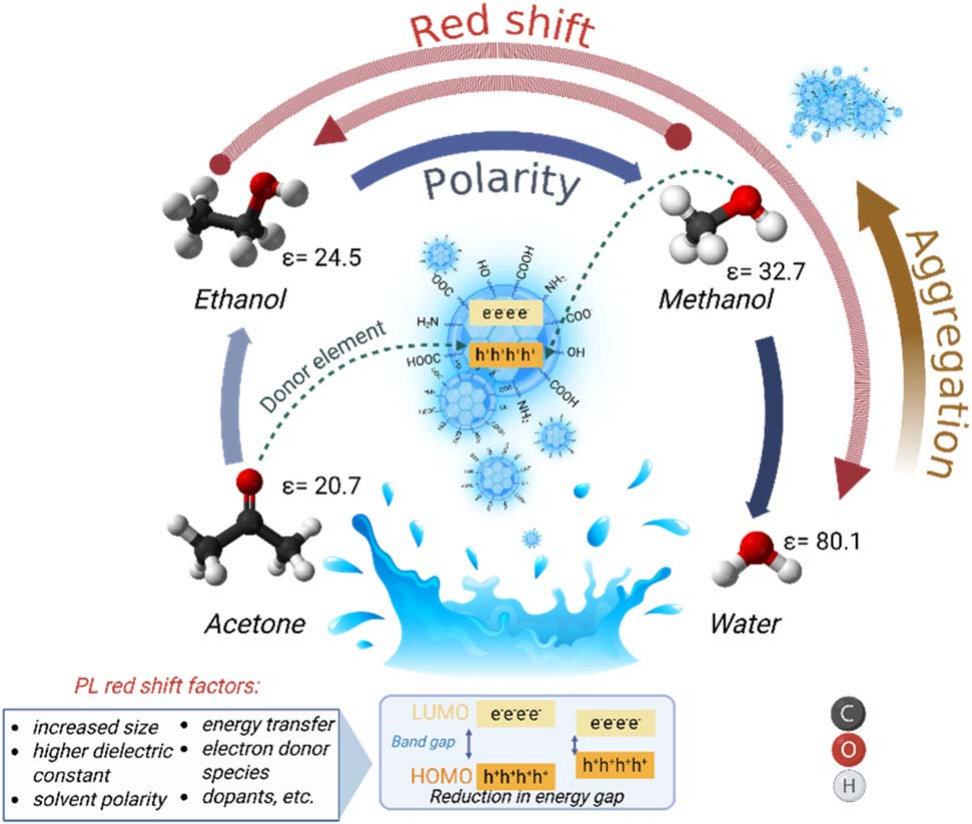
Schematic of the possible interactions of the CDs with different solvents.

The PL spectral variations observed for CDs derived from *E. crassipes* roots dispersed in different solvents (Fig. 3e and f) clearly demonstrate a solvent-dependent behavior. The solvent environment can significantly influence the PL emission of CDs through interactions with their surface functional groups, including hydrogen-bonding and dipole–dipole interactions, and internal charge transfer mechanisms, which can distort the electron density, amplifying the spectral shifts.^46^ In addition, the changes in the PL emission spectra when the CDs were dispersed in different solvents can be related to the disparity in the size distribution of the CDs.^47,48^ Stronger hydrogen bonds potentiate the aggregation of CDs, leading to multiple emissions.^14^ The neutral zeta potential measured for CDs-EtOH confirms their tendency to aggregate, in contrast to the zeta potential of CDs-w, which indicates good colloidal stability. An increased number of oxygen-containing functional groups (surface defects) can act as trapping sites for excitons, affecting the energy levels and potentially causing redshifts.^49,50^ As shown in the FTIR spectrum (Fig. 3a), the band corresponding to the C=O stretching vibrations is more pronounced in CDs-w compared to the peak observed for CDs-EtOH. In addition, the observed spectral differences in the ^1^H NMR analysis provide clear evidence of the influence of solvent environment on the structural features of the CDs (Fig. 4). Fig. 9 shows a schematic of the potential mechanisms responsible for the observed changes in the PL emission of the as-prepared CDs.

The advantages of CDs obtained from water hyacinth roots as reused biomass in comparison with other CDs or their composites are highlighted in Table 1.

**Table 1:** Comparison of CDs from different waste precursors (pollutant and non-pollutant) used for the detection of ethanol, methanol and acetone

Nanomaterial	Solvent detected	LOD (%v)	Linear concentration range (%v)	Number of responses			Ref.
				PL-turn-off (*λ*_em_)	PL-turn-on (*λ*_em_)	Other	
ZnS : Mn^2+^ quantum dot (QDs) and soluble *N*-methylpolypyrrole (NMPPy) hybrid	Methanol in water and ethanol	0.004	0.1–0.9	—	1 (∼422 nm)	—	51
CDs from 1-octyl-3-methylimidazolium tetrafluoroborate ([OMIM]BF_4_), *ortho*-phenylenediamine (*o*-PDA), and 1,3,5-benzenetricarboxylic acid (BTC)	Ethanol in water	0.12 and 0.16 by RGB analysis	0–100	—	1 (∼605/655 nm)	1 (RGB)	52
CDs from waste oil sulfur doped	Acetone in water	0.01	0.01–40	327 and 633 nm	411 and 764 nm	—	53
CDs from citric acid and urea	Methanol in water	0.025	0.0125–1	445 nm	—	—	54
	Alcoholic beverage, brand 1	0.75		425 nm			
	Alcoholic beverage, brand 2 alcoholic beverage, brand 3	0.18		425 nm			
	Alcoholic beverage, brand 4	0.42		425 nm			
		0.11		450 nm			
CDs from glucose and ammonium hydroxide	Acetone in water	0.09	0.1–0.5	1 (∼446 nm)	—	—	55
**CDs from water hyacinth roots**	**Acetone in water**	**0.03**	**0.05–0.33**	**2 (∼289/302 nm and ∼577 nm)**	**1 (+blue shift, ∼409 nm)**	—	**In this work**
	**Ethanol in water**	**1**	**1–37**	**0**	**2 (+red shift, ∼308 and ∼595 nm), 1 (+blue shift, ∼432 nm)**		
	**Methanol in ethanol**	**1.4**	**0.3–33**	**3 (364, 443 and 712 nm), 2 (+blue shift, 318 and 616 nm)**	**0**		
	**Methanol in vodka**	**1**	**1–45**	**0**	**2 (∼316 and ∼420 nm), 1 (+blue shift, ∼628 nm)**		

## 4. Conclusions

By simple carbonization of invasive *E. crassipes* roots harvested from a contaminated water lagoon, fluorescent and semicrystalline CDs were produced, highlighting a sustainable waste-to-resource strategy. The as-prepared CDs exhibited strong solvent-dependent optical properties, enabling their application in the multi-response detection of acetone and ethanol at trace concentrations in water. The ratiometric sensor, based on wavelength displacement and PL intensity, was demonstrated to be a viable strategy to detect methanol spiked in ethanol, and the method was validated through five verification points. These multi-emission responses of CDs provide the basis for an optical sensor with high accuracy, enhanced reliability, and reduced susceptibility to interference. The changes in the electronic structure generated by the solvation effect of CDs offer an effective, simple, and eco-friendly analytical approach for identifying and quantifying methanol in counterfeit and illicit alcoholic beverages, as well as determining ethanol and acetone concentrations in water.

## Author contributions

M. Rangel: conceptualization, data curation, formal analysis; investigation, methodology, validation, visualization, writing – original draft; S.D. Torres Landa: conceptualization, data curation, formal analysis, investigation, methodology, validation, visualization, writing – original draft; I. E. Serrato-Mireles: data curation, formal analysis, investigation, methodology; Y. Kumar: conceptualization, formal analysis; investigation, writing – original draft; N. Dasgupta Schubert: resources, supervision, writing – review & editing; J. E. García: resources, supervision, writing – review & editing; J. S. Pérez-Huerta: resources, supervision, writing – review & editing; and V. Agarwal: conceptualization, visualization, project administration, resources, supervision, writing – review & editing.

## Conflicts of interest

The authors declare no conflicts of interest.

## Supplementary Material

RA-016-D6RA00409A-s001

## Data Availability

The manuscript includes all relevant data that were generated or analyzed during this study. Additional information is included in the supplementary information (SI). Supplementary information: Table S1: physicochemical characteristics of water and *E. crassipes* roots from Jovita lagoon in “*Naranja de Tapia*”, Michoacán, México; Table S2: contaminants and micro/macro-nutrients detected by TXRF (total reflection X-ray fluorescence) in *E. crassipes* roots used as precursors for CDs and the Jovita lagoon water; Fig. S1: FESEM image of CDs-w dried on a silicon substrate and dried at 60 °C; Fig. S2: effect of excitation wavelength on the emission spectra of CDs-w; Fig. S3: influence of (a) NaCl concentration and (b) pH on the optical properties of CDs-w. See DOI: https://doi.org/10.1039/d6ra00409a.
