# Supplementary material for: Carbon dots from contaminated Eichhornia crassipes roots for spectrally multiplexed identification and sensing of solvents
Source: RSC Adv. 2026 Apr 28;16(24):22130–43. doi: 10.1039/d6ra00409a (PMC13122303; doi:10.1039/d6ra00409a)
Supplement: RA-016-D6RA00409A-s001 [file RA-016-D6RA00409A-s001.pdf]

Supplementary Information (SI) for RSC Advances.
This journal is © The Royal Society of Chemistry 2026

Supporting Information

Carbon Dots from Contaminated Eichhornia Crassipes Roots for Spectrally Multiplexed Identification and Sensing of Solvents

M. Rangel[1,2], S.D. Torres Landa[1], I. E. Serrato-Mireles[3], Y. Kumar[1,4], N. Dasgupta Schubert[3], J. E. García[5], J. S. Pérez-Huerta[2], V. Agarwal[1*]

[1] *Centro de Investigación en Ingeniería y Ciencias Aplicadas, IICBA- UAEM, Av. Univ. 1001, Col. Chamilpa, Cuernavaca, Morelos, 62209, México*
[2]*Unidad Académica de Ciencia y Tecnología de la Luz y la Materia-UAZ, Circuito Marie Curie S/N, Parque de Ciencia y Tecnología QUANTUM Ciudad del conocimiento, 98160, Zacatecas, Zac., México*
[3]*Facultad de Ciencias fisicomatemáticas, Universidad Michoacana de San Nicolás de Hidalgo, Santiago Tapia 403, col. Centro, 58000 Morelia, Mich., México*
[4]*Facultad de Física y Matemáticas (FCFM), Universidad Autónoma de Nuevo León (UANL), Cd. Universitaria, San Nicolás de Los Garza, N.L.,66451, México*
[5]*Secihti -Cinvestav, Unidad Saltillo, Ave. Industria Metalúrgica 1062, Parque Industrial, Ramos Arizpe, 25900, Coahuila, México*

Corresponding author e-mail: vagarwal@uaem.mx

Table S1. Parameters of water from Jovita lagoon in *"Naranja de Tapia"*, Michoacán, México and *E. crassipes* roots.

**Parameter**	**Polluted water**	***E. crassipes* roots**
pH	8.4 ± 0.15	7.14 ± 0.18
Conductivity (mS)	533.3 ± 4.73	4.87 ± 0.18
TDS (mg/l)	376.77 ± 1.07	3.39 ± 0.02
Nitrogen (mg/l)	-	9 ± 0.6
Phosphorus (mg/l)	0.0952 ± 0.0091 *	102.6 ± 1.2
Nitrite (mg/l)	0.0037 ± 0.0001 *	-
Nitrate (mg/l)	0.0185 ± 0 *	-
Orthophosphates (mg/l)	0.0524 ± 0.0036 *	-
Ammonium (mg/l)	0.0103 ± 0.0003 *	-
Total hardness +	169 ± 1.4142 *	-
Calcium hardness	73 ± 4.2426 *	-
Alkalinity (mg/l)	300 ± 2 *	-

TDS (Total dissolved solids)
*Determined according to APHA, AWWA & WEF test methods. Value parameters in accordance with NMX-001-SAGARPA-2021.

Table S2. Contaminants and micro/macro-nutrients detected by TXRF (Total reflection X-ray Fluorescence) in *E. crassipes* roots used as precursors for CDs and the Jovita lagoon water.

**Macronutrients**	**Polluted water (mg/l)**	***E. crassipes* roots (mg/l)**
Potassium	0.53 ± 0.16	116.09 ± 0.17
Calcium	13.25 ± 0.043	12.75 ± 0.035
Sulphur	8.65 ± 0.11	45.51 ± 0.16
Phosphorus	0.53 ± 0.16	11.60 ± 0.15
**Micronutrients**		
Iron	0.087 ± 0.003	0.127 ± 0.002
Zinc	0.031 ± 0.002	0.054 ± 0.001
Manganese	0.021 ± 0.004	0.006 ± 0.003
Copper	0.016 ± 0.002	0.064 ± 0.002
Chlorine	0.353 ± 0.051	8.746 ± 0.0062
Nickel	0.006 ± 0.002	0.022 ± 0.002

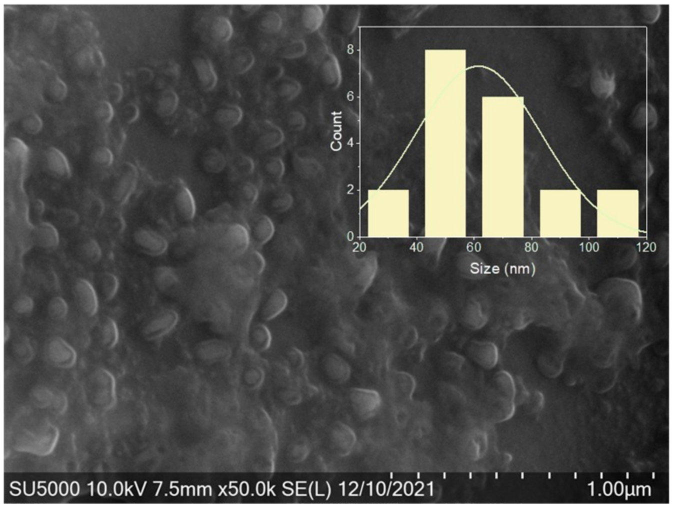

Figure S1. FESEM image of CDs-w dried on a silicon substrate and dried at 60°C.

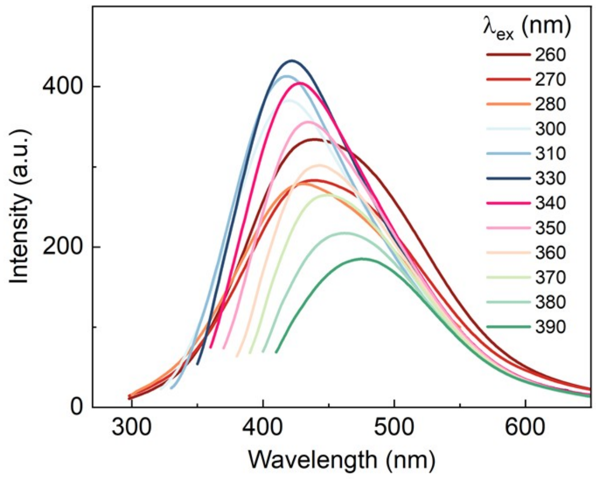

Figure S2. Effect of excitation wavelength on the emission spectra of CDs-w.

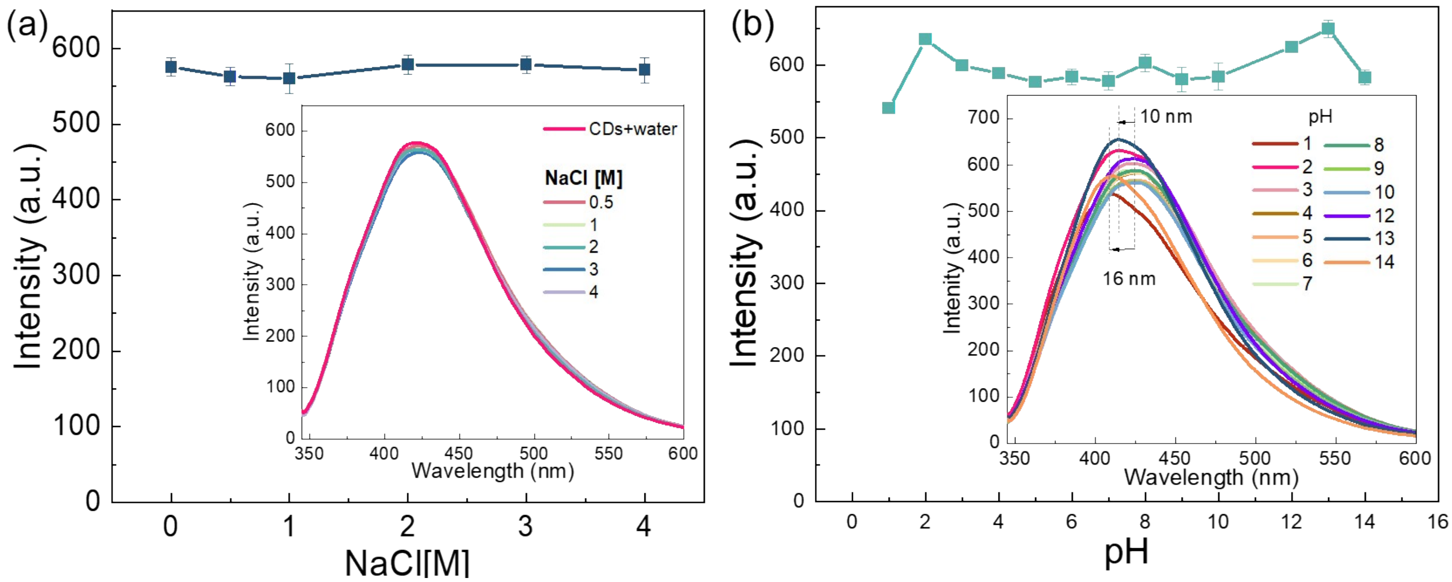

Figure S3. Influence of (a) NaCl concentration and (b) pH in the PL of CDs-w.
